# Fistula of the Cornea and Its Treatment

**Published:** 1875-06

**Authors:** A. W. Calhoun


### FISTULA OF THE CORNEA AND ITS TREATMENT.

BY A. W. CALHOUN, M.D.

*Read before the Atlanta Academy of Medicine May* 11, 1875.

By fistula of the cornea is meant a minute opening through the cornea into the anterior chamber, by means of which the aqueous humour is constantly discharged. Occasionally this takes place after injuries or operations, particularly when the latter is made in the limbus or corneo-sclerotic junction, with or without a conjunctival flap, for the conjunctiva heals over the wound, leaving in the limbus underneath, an unhealed point, through which the water is continually oozing, sometimes to be checked by the overlying conjunctival membrane.

Most frequently, however, the fistula is the result of a perforating corneal ulcer, which, when of the very smallest size and under most favorable circumstances, not unusually requires months and months to be brought to a perfect and permanent cure. The incessant outflowing of the aqueous humour, the adhesion of a part of the iris to a portion of the inner edge of the ulcer, and the constant pressure from the lens, which, meeting no resistance, bulges forward, all serve to keep up such a degree of irritation, that the fistulous track is prevented from healing.

The opening may, for the time being, become closed, and the aqueous humor may reaccumulate, but upon the least exertion by which the muscles of the eye are brought into play, or indeed without any assignable cause, the ulcer again ruptures, the aqueous flows off, and the anterior chamber in reality disappears. Even after an apparently final cure of the fistula, it may, and often does, again break open from the slightest causes which tend to irritate the eye. This tendency of the newly-formed corneal tissue to again give way and reopen the fistula, specially shows itself in those cases where the iris has become adherent to the posterior ulcerated surface of the cornea, in other words, to the edges of the perforation.

One case, and, so far as I know, the only one upon record, has been reported where the fistula occurred as a congenital malformation, inflammation and perforation of the cornea with failure to heal, having no doubt taken place *in utero*.

Not unfrequently fistula of the cornea becomes a most stubborn and dangerous disease, the constant external irritation leading, in many instances, to some deep-seated inflammation, which, continuing or recurring, at last ends in atrophy of the eye-ball. While the fistula exists, the eye is more or less red and irritable, the anterior chamber remains empty, and a drop of the aqueous humour is seen slowly exuding through the aperture and trickling down over the cornea. This may be more distinctly noticed by brushing away the fluid that has collected, by directing the patient to look slightly downward, and with the finger's end moving the lower lid to and fro over the cornea, and watching it recollect in the aperture.

Intra-ocular tension is of course greatly diminished, and should no occasional or temporary closure of the aperture take place, the ball sometimes becomes extremely soft, the vitreous becoming fluid and offering not even the slight resistance to the touch that is found in the sound vitreous.

A corneal fistula is oftener overlooked than one would imagine, the frequently recurring or continued trouble in the eye being attributed by the casual observer to some very obscure cause. Yet it bears certain characteristic appearances which often lead at once to their discovery. The orifice is a very minute, dark-colored spot, reminding one of the presence of a foreign body, such as a small piece of iron, in the cornea, and surrounding it is an opaque circle with a few capillary blood-vessels passing over it, up to its very inner edge and ending just at the dark spot. The little vessels frequently run together and form a sort of circle of loops around the opening. It is not invariable that these vessels are always present in any number, but almost always if the cornea has to a great extent, or entirely, become opaque; when also the circular opacity immediately around the fistula becomes more prominent and is seen to greater advantage. The obliteration of the anterior chamber, the fluid exuding from a certain spot and being brushed away, again exuding, the eye being in a constantly irritable condition, these, with the other appearances, will, without much difficulty, lead one to a correct diagnosis.

That the cure is difficult, is evidenced from the fact that innumerable modes of treatment have been suggested and practiced, and that still others are being daily offered. As a rule, the treatment for the fistula should, for a time at least, be a continuation of the same that was practiced for the healing of the corneal ulcer. It has been observed that a certain amount of traction upon the iris, particularly that portion which is adherent to the fistulous opening, has sometimes materially aided in effecting a cure, and with this end in view, both atropine and the extract of calabar bean have been used. The first tends to dilate the pupil, and hence exercises the traction of the muscular fibres, contracting from the pupillary margin towards the periphery of the iris. Where this fails, contraction in the opposite direction is attempted by the use of the latter remedy, (the Calabar bean) for this excites to action the sphincter muscle surrounding the pupil. The object of these two modes of treatment, acting apparently in opposite directions, is by the traction of the iris one way or the other, to set up sufficient inflammation around and in the aperture to effect its closure. Sometimes, also, it has been noticed that only after stopping the use of atropine, which had been continued uninterruptedly for a long time, has the fistula shown a disposition to heal. Possibly this took place by the same principle as above mentioned, being brought into play through the natural change in the condition of the iris. The atropine being left off, and the contraction of the muscular fibres following, the traction of the iris in the one direction is relieved, and, to some degree, set up in the other, by the natural contractility of the tissue, whereby a sufficient *adhesive inflammation*, so to speak, arises to close the fistula.

Under the belief that the fistula was due to an eversion of the internal lining membrane of the cornea, (the membrane of descemet) thus forming a short canal thoroughly lined with epithelium, it has been advised and successfully practiced in some instances, to grasp with a pair of forceps the wall of the canal or fistulous track, and bruise and tear its lining, thus ridding it of its epithelial layer. Then, with a compress and bandage, the eye is kept quiet, and in some instances good cures have been known to result. This is precisely the same principle as practiced in ordinary fistulous canals in any part of the body, where it is absolutely essential to first denude the inner surface of the canal of its epithelium, before anything like a cure can be looked for.

Touching the fistula with nit. silver, either in stick or solution, has been practiced, but it so often leaves behind a permanent cicatrix that other and less injurious modes of treatment are, as a rule, put into use.

But the surest of all the remedies—surer in closing up the aperture, and surer in warding off the most dreadful of the remote evil consequences of the disease (glaucoma)—is the iridectomy; and, to more fully illustrate its good effect, it will not be amiss to report the following case:

About one year previous to the time of presenting himself for treatment, Mr. C., æt. thirty, had had a severe attack of gonorrhœal ophthalmia in his left eye. The disease followed its usual course, resulting in the complete destruction of fully two-thirds of the cornea, leaving only a narrow margin at its periphery. The ulcerated surface gradually healed over, a new-formed tissue taking the place of the original corneal substance, and where the inflammation had entirely disappeared, the whole cornea presented a pearly, opaque appearance, utterly destroying all vision, a mere perception of light still existing. The patient came asking only for relief from the incessant escape of tears over the lower lid, the constant irritable and injected condition of the eye, and a dull, uneasy sensation within the ball. Examining the eye, there was the unnaturally congested condition of the conjunctival blood-vessels; the eye was filled with water as from excessive weeping, and in the centre of the opaque cornea there was a still more opaque ring, encircled by loops of minute vessels having in its centre a very small black spot, from which was exuding slowly but constantly the fluid from within the diminished or flattened anterior chamber. Rubbing the lid over the opening, and thus removing the water, it could be plainly noticed to again and again gather upon the black spot and drop down over the cornea into the lower conjunctival fold. The nature of the disease was easily recognized, and the patient advised to submit to an operation for its relief, to which he readily assented. Assisted by Drs. Baird, Kendrick, Todd and Ridley, the patient was anæsthetized, and, through an opening made at the upper corneo-sclerotic junction, a pair of forceps was passed down behind the iris until the points were behind the fistula, when as large a piece of the iris was withdrawn and clipped off as possible. This was done several times, each time bringing the instrument in contact with the posterior portion of the fistulous opening, thus irritating and denuding this part of the cornea as much as prudence would allow. The compress bandage was applied and allowed to remain four or five days, the eye being washed twice daily with simple cold water.

Upon removing the bandage the fistula was found to be thoroughly closed, no water was oozing out of it, the ball had assumed more of its natural tension, and the deep, dull pain had altogether vanished. The eye has remained in this condition up to this time, now nearly two months, and the irritability also no longer exists.

By means of the iridectomy we accomplish more than the relief of the fistula; we cut short the tendency of the eye to pass over into that glaucomatous condition, which is not a very unfrequent consequence of the disease, and which results in the loss of whatever vision may still exist, and often subjecting the patient to the most excruciating pains.

